# Associations between text communication engagement and maternal-neonatal outcomes in the Mobile WACh NEO Trial

**DOI:** 10.1371/journal.pdig.0000968

**Published:** 2025-08-07

**Authors:** James Peng, Erica Wetzler, Brenda Wandika, Peninah Kithao, June Moraa, Jenna I. Udren, Olivia Schultes, Esther Akinyi, Lusi Osborn, Anna Hedstrom, Barbra A. Richardson, Manasi Kumar, Dalton Wamalwa, John Kinuthia, Keshet Ronen, Jennifer A. Unger

**Affiliations:** 1 Department of Biostatistics, University of Washington, Seattle, Washington, United States of America; 2 Department of Global Health, University of Washington, Seattle, Washington, United States of America; 3 School of Nursing, University of Washington, Seattle, Washington, United States of America; 4 Department of Research and Programs, Kenyatta National Hospital, Nairobi, Kenya; 5 Department of Paediatrics-Neonatology, University of Washington, Seattle, Washington, United States of America; 6 Institute for Excellence in Health Equity, New York University Grossman School of Medicine, New York, New York, United States of America; 7 Department of Paediatrics and Child Health, University of Nairobi, Nairobi, Kenya; 8 Department of Obstetrics and Gynecology, Warren Alpert Medical School of Brown University and Women and Infants Hospital, Providence, Rhode Island, United States of America; The University of Hong Kong, HONG KONG

## Abstract

Despite a global reduction in neonatal deaths in the last few decades, high neonatal mortality rates persist in low- to middle-income countries. Mobile health interventions offer a promising solution to promote early newborn care (ENC) practices and improve neonatal health. The Mobile WACh NEO randomized controlled trial evaluated the effect of a text messaging communication intervention on neonatal health outcomes in Kenya from 2020 to 2023. Perinatal participants received automated messages from enrollment at 28–36 weeks gestation until six weeks postpartum and could message with a study nurse. This secondary analysis aimed to characterize participant text engagement and examine associations between engagement and maternal-neonatal health outcomes. Among 2,470 intervention participants retained through follow-up, median time in the intervention was 14 weeks. Participants received a median of 58 automated messages (average 0.58 per day), sent a median of 24 messages (average 0.25 per day), and received a median of 14 nurse responses (average 0.14 per day). Younger, more educated, unmarried, unemployed, and first-time mothers sent more messages, while those who had a lower social support score at baseline messaged less. Increased participant messaging was associated with greater increase in neonatal danger sign knowledge from baseline to six-week follow-up (Adj Est: 0.39; 95% CI: 0.09-0.68) and lower odds of early initiation of breastfeeding (aOR: 0.62; 95% CI: 0.45-0.86). Our findings contribute to the understanding of who can benefit from mobile health programs and how these interventions might impact behaviors and outcomes.

## Author summary

In this study, we examined how engagement with a mobile health intervention — designed to support mothers during pregnancy and early motherhood— was related to maternal and infant health outcomes. The intervention provided automated health information and allowed mothers to communicate directly with nurses via text messages. Younger, more educated, unmarried, unemployed, and first-time mothers engaged with messaging more, while those with less social support messaged less. We found that higher levels of engagement were linked to improved knowledge of neonatal danger signs, but also to lower odds of early breastfeeding initiation. Although we are unable to conclude that messaging directly impacted any health outcomes, our findings suggest that mothers who engaged more may have sought additional support. This work contributes to ongoing efforts to optimize mobile health programs, particularly in resource-limited settings, by offering insights into engagement patterns and how they might mediate a program’s effectiveness. Our study provides an example of how engagement in mHealth interventions can be reported in relation to health outcomes. We hope this work inspires other researchers to explore which participants engage with their mobile health programs and who stands to benefit most from these interventions, guiding the design and implementation of more effective programs.

## Introduction

Despite improvements in global neonatal mortality rates over the past few decades, decreasing by 52% from 1990 to 2019, high newborn mortality persists in many parts of the world [1]. For example, southern and eastern Africa had a neonatal mortality rate of 23.8 deaths per 1,000 live births in 2019, compared to 3.7 deaths per 1,000 live births in North America. The neonatal mortality rate in many countries significantly surpasses the Sustainable Development Goals target of 12 per 1,000 live births by 2030 [2]. Most neonatal deaths can be averted with access to evidence-based, cost-effective interventions, such as early newborn care (ENC) practices of early and exclusive breastfeeding, clean cord care, thermal care, and kangaroo mother care (KMC) for preterm and low birthweight infants [3–6]. ENC practices have been shown to reduce mortality and improve infant health outcomes, yet they remain underutilized in sub-Saharan Africa [7–9]. Additionally, educating mothers to recognize neonatal danger signs (NDS) fosters timely care-seeking in critical situations [10]. Finally, as prenatal and postnatal mental health disorders affect approximately one in five mothers, and are linked to infant survival and development outcomes, providing psychosocial support to mothers is another effective preventive strategy [11,12].

Mobile health (mHealth) interventions - which utilize mobile devices to support medical and public health practice - are promising tools to promote maternal ENC practices, increase maternal awareness of neonatal danger signs, encourage active care engagement, and enhance maternal psychosocial well-being [13]. Patient-directed mHealth interventions often aim to drive behavior change by delivering information, strengthening motivation to engage in health behavior, and offering psychosocial support to empower patients [14]. The recent spread of mobile technologies have made mHealth interventions increasingly feasible and acceptable [15]. In Kenya in particular, over 90% of the population has access to a mobile phone, and 92% of those with a phone said they would like to receive weekly SMS messages from their healthcare provider [16]. Text messaging communication interventions have been used to improve clinic attendance, increase contraceptive use, encourage breastfeeding practices, and reduce mother-to-child transmission of HIV with varying degrees of success [17–21]. The Kenya Ministry of Health has increasingly embraced the use of mHealth as part of health service delivery [22].

When implementing mHealth interventions, assessing participant engagement - how much users interact with the innovation - is crucial for interpreting their effectiveness, understanding the mechanisms driving behavior change, and identifying which groups benefit the most from the intervention [23]. Research on engagement in mHealth interventions has shown that engagement levels vary by approach, communication modality, and setting [24–27]. Some studies have explored associations between engagement and health outcomes and found a dose-response relationship [28,29]. Literature specifically addressing engagement in text messaging in resource-limited settings is particularly sparse, with most studies reporting only response rates and basic engagement outcomes [18,30,31]. We identified only one study in a related setting that examined how engagement with a SMS intervention varies across participant characteristics [31]. Additionally, to our knowledge, no published work has yet explored associations between text messaging engagement and maternal-neonatal health outcomes.

The Mobile WACh NEO study was a two-armed randomized controlled trial conducted among peripartum women in Kenya from September 2020 to February 2023. The study aimed to evaluate the efficacy of a bi-directional SMS messaging intervention in reducing neonatal mortality, with secondary outcomes including the adoption of ENC practices, infant hospitalization, and maternal mental health [32]. The primary analysis revealed no significant differences in outcomes between the intervention and control groups [33]. The purpose of this secondary analysis is twofold. First, we aim to characterize participant messaging engagement among intervention participants, both overall and stratified by baseline characteristics. Second, we aim to analyze associations between messaging behavior and maternal-neonatal health outcomes, including neonatal mortality, maternal adoption of ENC practices, and maternal psychosocial outcomes. With these two aims, we seek to characterize who was most likely to engage in the SMS intervention and analyze whether increased engagement was associated with better maternal-neonatal outcomes.

## Methods

### Mobile WACh NEO

The Mobile WACh NEO study was a multisite, two-arm randomized controlled trial (RCT) comparing a text messaging intervention to standard care (no messaging). The protocol was approved by ethics committees at the University of Washington, Women and Infants Hospital, and Kenyatta National Hospital/University of Nairobi. Eligible participants provided written informed consent after completing the informed consent process. The age of consent in Kenya is 18, but pregnant women aged 14 or older were considered emancipated minors and thus consented independently. Details of the RCT are described in the protocol publication [32].

Briefly, participants in six health facilities in Kenya (two in peri-urban settlements of Nairobi and four in Western Kenya) were individually randomized to the intervention group to receive a series of automated SMS messages tailored to their gestational age or postpartum period, delivered from enrollment (28–36 weeks gestation) until six weeks postpartum. These messages provided anticipatory guidance, support for essential newborn care (ENC) practices, and screening for neonatal danger signs, promoting timely care-seeking behaviors. Messaging frequency varied throughout the intervention, with the most frequent messaging happening around the peripartum period (i.e., two weeks before the due date and two weeks postpartum) to capture information on deliveries as well as screen for urgent NDS. Participants could send messages with questions and concerns, free of charge, to the system at any time. Messages were responded to by one of 12 licensed Kenyan nurses, employed by the study, with training in maternal, infant and child health. Typically, two nurses were stationed at each of of the six study sites and responded to messages from participants at their site during normal business hours, triaging urgent cases as needed. Nurses received additional training on all study topics, as well as responding to health-related text messages. All staff received ongoing supportive supervision and refresher trainings through weekly message reviews by senior nurse coordinators, obstetrician-gynecologist, and neonatologist study team investigators. We refer to three types of messages in upcoming sections: system automated, participant, and nurse messages.

The intervention included adaptations for high-risk mother–infant dyads, who were placed in specific messaging tracks. High-risk groups included first-time mothers or mothers age ≤ 19, women exhibiting elevated depression symptoms (Edinburgh Postnatal Depression Scale score ≥13) [34], and mothers of premature (<37 weeks gestation) or low birthweight (<2.5 kg) infants. These participants received targeted messages that provided specific guidance and support relevant to their risk factors. If a participant experienced pregnancy loss, stillbirth, or infant death and consented to continue receiving messages, the content shifted to provide emotional support and condolence messages for four weeks, excluding any pregnancy or infant care advice. The five messaging tracks were (1) general, (2) first-time mother, (3) preterm/low birth weight, (4) depression, and (5) infant loss. Study nurses switched participants between messaging tracks based on assessments for risk factors during study data collection.

Maternal-neonatal outcomes were collected through text communication and at fixed visits at two weeks and six weeks postpartum. These included neonatal mortality (death in first 28 days of life), initiation of early breastfeeding (breastfeeding in first hour of life), exclusive breastfeeding (during first six weeks of life), appropriate thermal care (no bath in first 24 hours of life), home provision of kangaroo mother care (KMC) (skin-to-skin care on ≥10 of the first 14 days at home, among low birthweight or preterm infants), cord care (no application of substances to cord), and infant hospitalization (within first six weeks). Additionally, study staff ascertained depression symptoms (Edinburgh Postnatal Depression Scale), social support score (Medical Outcomes Study [MOS] Social Support Survey), self-efficacy score (Karitane Parenting Confidence Scale), and knowledge of 8 neonatal danger signs at the enrollment, two week, and six week visits [33–35].

### Study population

In this secondary data analysis, we included only intervention participants who received at least one message and completed their six week postpartum study visit. For mothers who gave birth to twins, only the firstborn was considered in this analysis.

### Characterizing participant engagement

To quantify overall participant engagement, we calculated system automated, participant, and nurse messages and message character count in total and averaged each of these data points over the number of days the participant was in the study. Additionally, we present average weekly message counts relative to the delivery date and the proportion of participant messages that study nurses classified into topic categories, including general infant health, antenatal concerns, essential newborn care, breastfeeding, delivery concerns, and others.

We examined associations of participant messages with each of the following baseline characteristics: age group (age 14–19, 20–24, 25–29, and 30 or older), education (secondary education or no secondary education), marital status (married/cohabiting or not), primigravida (first pregnancy or not), intimate partner violence in the last month (yes/no), household crowding (more than three people per room in the house), depression score (by tertile), social support score (by tertile), and self-efficacy score (by tertile). We regressed normalized participant messages on each baseline characteristic using linear regression, adjusted by clinic, to obtain estimates and 95% Wald confidence intervals (from robust standard errors) for the differences of participant message count means in each category relative to reference categories.

### Association between messaging engagement and maternal-neonatal outcomes

#### Exposures.

Our primary measure of engagement is the average number of participant messages to the study nurse per day in the corresponding perinatal period (i.e., number of messages divided by the number of days in the period). We use period-specific exposures relevant to the outcome being measured. For example, only antenatal messages were considered for the early breastfeeding outcome, as this outcome was assessed immediately following the baby’s birth. Normalized messages were used instead of raw message counts to account for the varying amounts of time participants had to engage with the intervention. For the neonatal mortality outcome, we used antenatal messaging as the exposure, as 72% of infant deaths occurred during the first week after delivery. For the hospitalization outcome, we used normalized messages prior to hospitalization (for those whose infant was hospitalized) and messages across the entire period (for those whose infant was not hospitalized).

#### Outcomes.

We examined the binary outcomes of infant mortality, infant hospitalization, initiation of early breastfeeding, exclusive breastfeeding, appropriate thermal care, home provision of KMC among low birthweight or preterm infants, and appropriate cord care. Additionally, we examined continuous outcomes of changes in maternal knowledge of neonatal danger signs, maternal depression scores, social support levels, and self-efficacy scores from enrollment to six weeks postpartum. In S1 Table, we display a brief description of the outcomes, timing of the exposure, inclusion criteria, and the analytic approach taken.

#### Statistical analysis.

For binary outcomes, we employed logistic regression of the outcome on the period-specific exposure, adjusted by age, education, marital status, clinic, and primigravida. These adjustment variables were chosen due to their potential influence on both engagement with messaging and the outcome. For binary outcomes with <15 events (e.g., home provision of KMC), we report unadjusted estimates. The fitted coefficient obtained from these regressions can be interpreted as an odds ratio comparing the odds of the event for two groups with the same adjustment characteristics differing in one message sent per day. For continuous outcomes, we employed adjusted linear regression of the change in the outcome from baseline to six-week follow-up on the exposure. The resulting coefficient can be interpreted as the difference in the change in outcome from baseline for every 1-unit difference in the normalized messages. For outcomes with >10% missingness (early breastfeeding and self-efficacy), we performed multiple imputation to obtain estimates and confidence intervals. Statistical significance was assessed at the 0.05 level. Although multiple regressions were performed, we considered this an exploratory analysis, and we did not perform multiplicity adjustments. All statistical analyses were performed using R software (version 4.2.1).

#### Subgroups.

For outcomes showing a significant association in the entire study population, we performed a subgroup analysis in the following groups: preterm births and low birthweight infants, first-time mothers, and mothers classified at low-risk (e.g., no preterm birth, not a first-time mother, and no elevated depressive symptoms at baseline). These subgroups were considered in order to measure associations within groups with inherently different risks and behaviors. Note that a participant can be included separately in both the preterm/low birthweight birth and first-time mother subgroups. Mothers showing elevated depressive symptoms at baseline were initially considered a high-risk subgroup; however, due to the small sample size, we did not conduct a subgroup analysis on this group.

#### Sensitivity analysis.

We conducted the following sensitivity analyses: (1) unadjusted analysis and (2) using combined participant and nurse normalized message character count as the exposure. Our goal for (2) was to account for the length of both participant messages and nurse responses as proxies for the quality of participant and nurse reciprocal engagement.

## Results

### Participant characteristics

Of the 2,505 participants enrolled into the intervention arm, 2,470 (98.6%) received at least one message, were retained until study endpoint at six weeks postpartum, and therefore included in the analysis. Participants were in the study for a median of 14 weeks (IQR: 12–16). More than 99% (2,459/2,470) of participants indicated that they could read and write SMS messages unassisted. Baseline characteristics are shown in Table 1. Participants from Nairobi constituted 47.3% of the study population, with the remaining participants attending clinics in Western Kenya. The median age of women at enrollment was 25 years old (IQR: 22–29), 1,456 (58.9%) had a secondary education, 391 (15.8%) experienced household crowding, 1,974 (79.9%) were married, and 934 (37.8%) were employed. Eight hundred and fifty-two (34.5%) participants had first-time pregnancies, 76 (3.1%) had elevated depressive symptoms, and 89 (3.6%) experienced intimate partner violence in the last month. The median depression score at baseline was 1 (IQR: 0–3), social support transformed score was 80 (IQR: 66–100), self-efficacy score was 45 (IQR: 41–45), and NDS identification score was 2 (IQR: 1–3).

**Table 1:** Baseline characteristics of participants included in the analysis, overall and stratified by messaging track. For the first-time, depression, preterm/low birthweight, and infant loss messaging tracks, the numbers represent those who were at some point during the study in the messaging track. For the general messaging track, the numbers represent those who were *only* in the general messaging track throughout the study period. Participants could be counted in more than one track.

		Specialized track				Overall (N=2470)
	General (N = 1267)	First-time mother (N = 863)	Depression (N = 103)	Preterm/low birth weight (N = 349)	Infant loss (N = 69)	
**Nairobi clinic**	605 (47.8%)	419 (48.6%)	37 (35.9%)	146 (41.8%)	37 (53.6%)	1168 (47.3%)
**Age - Median (IQR)**	27 (24, 31)	21 (19, 23)	24 (21, 30)	24 (21, 29)	27 (23, 30)	25 (22, 29)
**Age Group**						
14-19	0 (0%)	224 (26.0%)	17 (16.5%)	42 (12.0%)	5 (7.2%)	239 (9.7%)
20-24	319 (25.2%)	491 (56.9%)	36 (35.0%)	135 (38.7%)	22 (31.9%)	909 (36.8%)
25-29	508 (40.1%)	123 (14.3%)	24 (23.3%)	89 (25.5%)	22 (31.9%)	741 (30.0%)
30+	440 (34.7%)	25 (2.9%)	26 (25.2%)	83 (23.8%)	20 (29.0%)	581 (23.5%)
**Has secondary education**	668 (52.7%)	633 (73.3%)	44 (42.7%)	197 (56.4%)	36 (52.2%)	1456 (58.9%)
**Experiences household crowding**	257 (20.3%)	65 (7.5%)	22 (21.4%)	56 (16.0%)	10 (14.5%)	391 (15.8%)
**Married/cohabiting** (n = 2,465)	1177 (92.9%)	509 (59.0%)	65 (63.1%)	263 (75.4%)	56 (81.2%)	1974 (79.9%)
**Employed** (n = 2,468)	598 (47.2%)	212 (24.6%)	25 (24.3%)	120 (34.4%)	22 (31.9%)	934 (37.8%)
**First-time pregnancy**	0 (0%)	828 (95.9%)	31 (30.1%)	128 (36.7%)	26 (37.7%)	852 (34.5%)
**Gestational age at delivery - Median (IQR)**	40 (39, 41)	40 (38, 41)	40 (38, 41)	36 (35, 37)	39 (37, 41)	40 (38, 41)
**Social support score - Median (IQR)** (n = 2,468)	78 (63, 100)	86 (72, 100)	63 (50, 80)	83 (68, 100)	82 (63, 100)	80 (66, 100)
**Self efficacy score - Median (IQR)** (n = 2,091)	45 (42, 45)	44 (39, 45)	43 (37, 45)	45 (42, 45)	44 (40, 45)	45 (41, 45)
**Depression score - Median (IQR)** (n = 2,464)	1.0 (0, 3.0)	1.0 (0, 3.0)	14 (13, 17)	1.0 (0, 3.0)	1.0 (0, 5.0)	1.0 (0, 3.0)
**Elevated depression symptoms (Score ≥ 13)**	0 (0%)	2 (0.2%)	76 (73.8%)	8 (2.3%)	5 (7.2%)	76 (3.1%)
**Maternal knowledge of NDS - Median (IQR)**	2.0 (1.0, 3.0)	1.0 (0, 2.0)	2.0 (1.0, 3.0)	2.0 (1.0, 3.0)	2.0 (1.0, 3.0)	2.0 (1.0, 3.0)
**Any intimate partner violence** (n = 2,408)	43 (3.4%)	14 (1.6%)	27 (26.2%)	8 (2.3%)	3 (4.3%)	89 (3.6%)

### Messaging tracks

**Fig 1:**
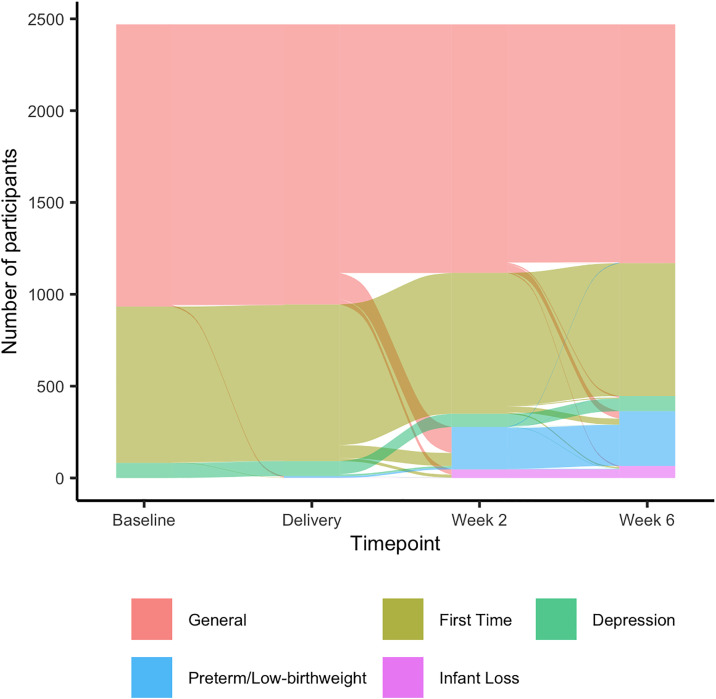
Flow diagram of the distribution of mothers through different messaging tracks over the course of the study.

We present the progression of participants through tracks in Fig 1. At baseline, 1,537 (62%) of participants were in the general track, 851 (35%) in the first-time mother track, and 82 (3%) in the depression track. At the end of their time in the intervention, 66 (3%) were in the infant loss track, 298 (12%) were in the preterm/low birthweight birth track, 82 (3%) were in the depression track, 724 (29%) were in the first-time mother track, and 1,300 (53%) were in the general track. Baseline characteristics stratified by track are shown in Table 1. Participants in the first-time/adolescent mother track were generally younger, with a median age of 21 versus 25 years in the overall cohort. A higher proportion had completed secondary education (73% compared to 59% of all participants), and fewer were married (59% compared to 80% overall). In the depression track, 63% of participants were married and 24% were employed, compared to 80% married and 38% employed in the overall group. Additionally, 26% in the depression track reported experiences of intimate partner violence, while this was reported by 4% of the overall cohort.

### Messaging engagement

**Fig 2:**
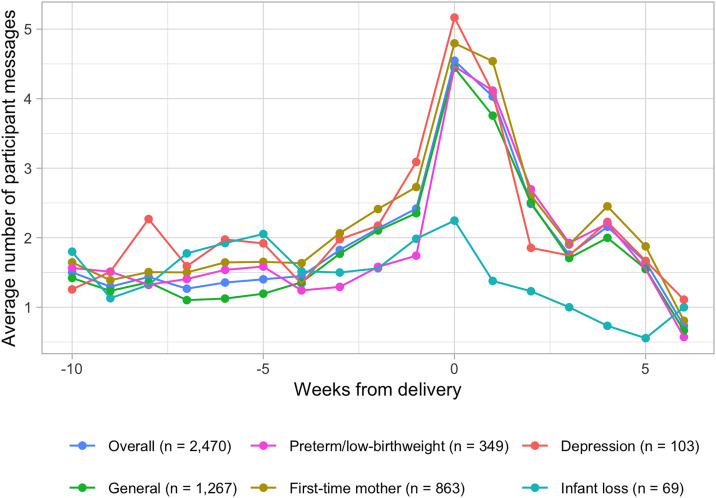
Average number of participant messages sent by week, relative to the date of delivery.

System automated, participant, and nurse message counts and lengths are shown in Table 2. Over the study period, participants received a median of 58 (IQR: 48–66) system automated messages, or 0.58 per day on average (IQR: 0.5 - 0.7). Participants sent a median of 24 (IQR: 9–44) messages total to study nurses, or approximately 0.25 (IQR: 0.1 - 0.46) per day on average. Overall, 2,287 (92.6%) of participants sent at least one message during the study period. The most common topics for participant messages were antenatal concerns (15.2% of messages sent), general infant health (14.5%), appreciation/acknowledgement (9.8%), essential newborn care (8.4%), breastfeeding (6.5%), delivery concerns (6.1%), and social support (5.3%). Study nurses sent a median of 14 messages (IQR: 5–26) per study participant, or approximately 0.14 (IQR: 0.05 - 0.27) per day. The average number of messages sent by participants per week, relative to the date of the delivery, are shown in Fig 2. From 10 weeks until two weeks prior to delivery, participants sent on average 1.6 messages per week (IQR: 0–2). During the week prior to delivery, participants sent on average 2.4 messages (IQR: 0–3). Participant messaging increased during the two weeks immediately following delivery to 4.5 messages during the week of delivery (IQR: 0–7) and 4.0 messages during the week after delivery (IQR: 0–6). Messaging declined to an average of 1.8 messages (IQR: 0–3) from two weeks after delivery until the end of the study. Participant messaging by week generally mirrored the frequency of system-automated and nurse messages throughout the study. The weekly average of system-automated and nurse messages is presented in S1 Fig.

**Table 2:** Median (IQR) number of messages and message characters sent (system automated, participant, and nurse messages) throughout the course of the study, in total and normalized by time in study.

	Median total number of messages sent (IQR)	Median daily messages (IQR)	Median total number of characters (IQR)	Median daily characters (IQR)
**System automated messages**	58 (48, 66)	0.58 (0.5, 0.68)	17,776 (14,401, 20,232)	178.0 (152.1, 207.9)
**Messages from participants**	24 (9, 44)	0.25 (0.1, 0.46)	699 (243, 1,443)	7.4 (2.6, 14.9)
**Messages from nurses**	14 (5, 26)	0.14 (0.05, 0.27)	1,403 (463, 2,742)	14.6 (4.9, 28.7)

Normalized messages across baseline characteristics are shown in Table 3. A clear age-related trend was observed, with younger participants sending more messages per day on average; participants in the same clinic under the age of 19 years sent on average 0.1 more messages per day (95% CI: 0.05-0.15, p < 0.001) compared to those who were aged 30 years or older. Those with a secondary education sent on average 0.08 more messages per day (95% CI: 0.06 - 0.10, p < 0.001) than those without, unmarried participants sent on average 0.06 more messages per day (95% CI: 0.03 - 0.09, p < 0.001) than those married, first-time mothers sent 0.06 more messages per day (95% CI: 0.03 - 0.08, p < 0.001) than non-first-time mothers, and those unemployed sent 0.04 more messages per day than those employed (95% CI: 0.01 - 0.06, p = 0.002). Those with the highest tertile of social support score sent on average 0.06 messages more per day than those in the lowest tertile (95% CI: 0.04 - 0.09, p < 0.001).

**Table 3:** Mean (SD) of normalized participant messages by baseline characteristics. The table shows estimates and confidence intervals from a clinic-adjusted linear regression, where normalized participant messages are regressed on baseline characteristics. For clinic comparisons, unadjusted results are shown, and the p-value shows the result of the ANOVA test assessing equality of mean messages between clinics.

Characteristic	Mean normalized messages (sd)	Estimated difference in mean from reference category	95% CI	p-value
Clinic (unadjusted)				<0.001
Clinic 1	0.23 (0.21)	—	—	—
Clinic 2	0.31 (0.29)	0.08	0.04, 0.12	
Clinic 3	0.32 (0.29)	0.09	0.04, 0.14	
Clinic 4	0.35 (0.29)	0.12	0.08, 0.16	
Clinic 5	0.29 (0.29)	0.06	0.02, 0.11	
Clinic 6	0.33 (0.29)	0.10	0.06, 0.14	
Age Group				
14-19	0.37 (0.34)	—	—	—
20-24	0.33 (0.30)	-0.05	-0.10, 0.00	0.03
25-29	0.31 (0.28)	-0.07	-0.12,-0.03	<0.001
30+	0.28 (0.24)	-0.10	-0.15, -0.05	<0.001
Education				
No secondary education	0.26 (0.27)	—	—	—
Secondary education	0.35 (0.30)	0.08	0.06, 0.10	<0.001
Household crowding				
No	0.32 (0.29)	—	—	—
Yes	0.31 (0.26)	-0.02	-0.05, 0.01	0.14
Marital status				
Not married	0.36 (0.34)	—	—	
Married	0.31 (0.27)	-0.06	-0.09, -0.03	<0.001
Employment				
No employment	0.33 (0.30)	—	—	—
Employed	0.29 (0.26)	-0.04	-0.06, -0.01	0.002
Primigravida				
No	0.30 (0.26)	—	—	—
Yes	0.35 (0.32)	0.06	0.03, 0.08	<0.001
Depression				
No	0.32 (0.29)	—	—	—
Yes	0.32 (0.26)	0.02	-0.04, 0.08	0.54
Any intimate partner violence				
No	0.32 (0.29)	—	—	—
Yes	0.30 (0.24)	-0.01	-0.06, 0.04	0.58
Social support score tertile				
1	0.28 (0.25)	—	—	—
2	0.32 (0.29)	0.05	0.02, 0.08	<0.001
3	0.35 (0.31)	0.06	0.04, 0.09	<0.001
Self-efficacy score tertile				
1	0.32 (0.29)	—	—	—
2	0.34 (0.30)	0.01	-0.02, 0.04	0.48
3	0.29 (0.27)	-0.01	-0.04, 0.03	0.68
Depression score tertile				
1	0.33 (0.30)	—	—	—
2	0.31 (0.29)	0.00	-0.03, 0.04	0.95
3	0.30 (0.27)	-0.02	-0.05, 0.01	0.30
NDS score				
0-1	0.33 (0.30)	—	—	—
2-3	0.31 (0.27)	-0.01	-0.03, 0.02	0.70
4+	0.29 (0.31)	-0.01	-0.05, 0.03	0.61

### Maternal-neonatal outcomes

S2 Table presents overall maternal-neonatal outcomes. Outcomes were ascertained for over 90% of participants, with the exception of early breastfeeding (85.4%) and change in self-efficacy score (77.9%), due to non-response. Among participants who met the inclusion criteria and had the respective outcomes ascertained, 1.8% of infants died within 28 days, 4% were hospitalized, and 72.4% mothers initiated early breastfeeding. Additionally, 91.8% of mothers exclusively breastfed during the study period, 96% of mothers practiced appropriate thermal care within the first 24 hours of birth, and 47% of mothers performed correct cord care. Of the mothers of preterm or low birthweight infants who met the inclusion criteria for the KMC outcome, 6.4% performed KMC care with their infants during ≥10 of the first 14 days at home. Mean changes in each score variable from baseline to six weeks are shown in S2 Table.

### Association between engagement and maternal-neonatal outcomes

**Fig 3:**
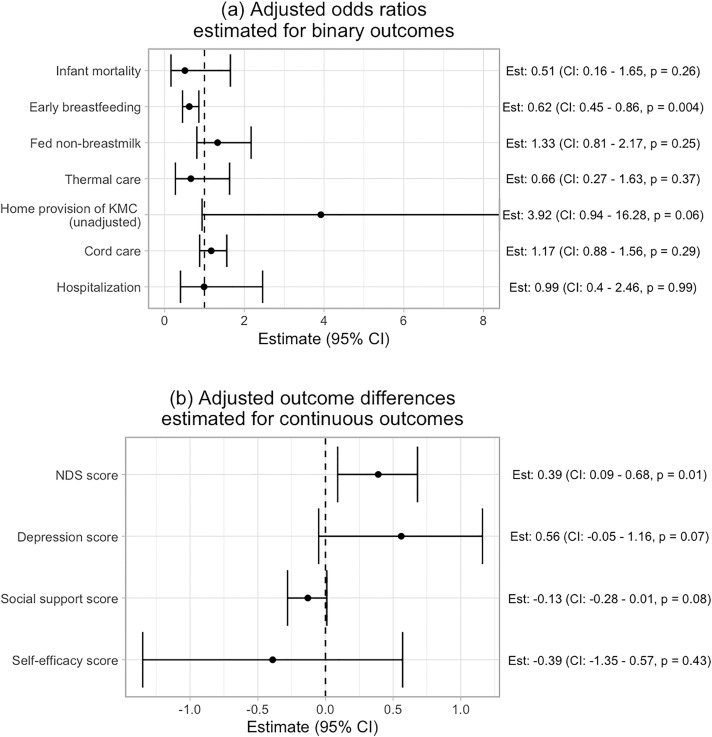
Association between number of participant messages sent and trial outcomes. **(a)** The top panel displays the estimated adjusted odds ratios when regressing each binary outcome on the normalized period-specific message count. **(b)** The bottom panel presents the estimated adjusted outcome differences when regressing the change in each score from baseline to 6-weeks on the normalized period-specific message count. For the home provision of KMC outcome, the unadjusted estimate is shown because only 11 participants had the positive outcome. For early breastfeeding and self-efficacy outcomes, multiple imputation was used to generate the estimates and confidence intervals due to missing outcome data.

**Fig 4:**
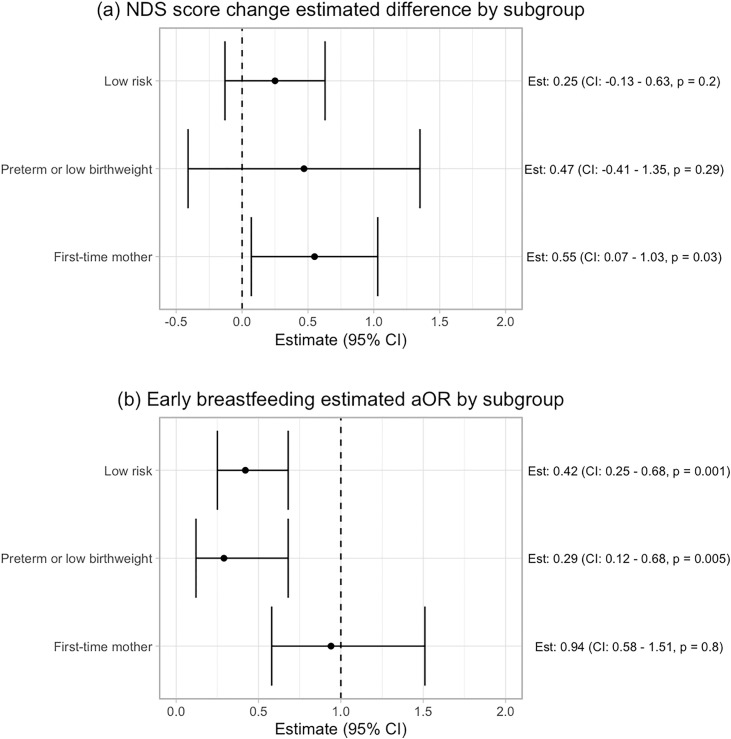
Results of association analyses by subgroups (low-risk, preterm/low birthweight, first-time mother) for (a) NDS score change outcomes and (b) early breastfeeding. Multiple imputation was used to obtain the early breastfeeding results.

Fig 3 summarizes the association between number of participant messages sent daily and maternal infant outcomes after adjusting for clinic, age, secondary education, marital status, and primigravida. Increased messaging by participants was associated with a greater increase in NDS knowledge score over the study period, with an average increase of 0.39 more danger signs identified from baseline to six-week follow-up for every additional message sent per day (95% CI: 0.09 - 0.68, p = 0.01). In subgroup analyses (Fig 4a), this difference was significant in the first-time mother group (Adjusted Est: 0.55, 95% CI: 0.07 - 1.03, p = 0.03), but not in the preterm birth (Adjusted Est: 0.47, 95% CI: -0.41 - 1.35, p = 0.29) and no risk groups (Adjusted Est: 0.25, 95% CI: -0.13 - 0.63, p = 0.2).

More participant messaging was significantly associated with a lower odds of early breastfeeding (Fig 3), with 38% lower odds of early initiation of breastfeeding for every additional message sent per day (aOR: 0.62, 95% CI: 0.45 - 0.86, p = 0.004). In subgroup analyses (Fig 4b), this association was significant in the preterm/low birthweight (aOR: 0.29, 95% CI: 0.12 - 0.68, p = 0.005) and low-risk groups (aOR: 0.42, 95% CI: 0.25 - 0.68, p = 0.001), but not in the first-time mother group (aOR: 0.94, 95% CI: 0.58 - 1.51, p = 0.8).

Although the association was not statistically significant, we estimated 3.92 times higher odds of providing KMC at home for each additional message participants sent per day among preterm and low birthweight infants (OR: 3.92, 95% CI: 0.94-16.28, p = 0.06). Additionally, we observed non-significant trends of associations between more messaging and greater increase in depression score over the study period (Adjusted Est: 0.56, 95% CI: -0.05-1.16, p = 0.07) and decrease in social support score (Adjusted Est: -0.13, 95% CI: -0.28-0.01, p = 0.08). Participant messaging behavior was not significantly associated with other outcomes - including neonatal mortality, exclusive breastfeeding, thermal care, cord care, and change in self-efficacy score. Sensitivity analyses showed similar results (S2 and S3 Figs).

## Discussion

The Mobile WACh NEO study aimed to engage peripartum women with healthcare services by enhancing maternal-neonatal health knowledge, improving essential newborn care skills and providing psychosocial support through automated informational text messages and providing access to study nurses for questions and concerns. We found that younger, more educated, unmarried, unemployed, first-time mothers and mothers with higher baseline social support tended to engage in more messaging with the study nurses. More frequent messaging was associated with increases in neonatal danger sign knowledge and lower odds of early breastfeeding. We also observed non-significant trends of higher messaging frequency association with a greater likelihood of home-based KMC, as well as increased depression scores and decreased social support scores.

Participant characteristics associated with engagement in the intervention largely align with our expectations. Younger and more educated mothers may have greater familiarity and comfort with mobile phone use, and unemployed participants likely have more flexibility with their time to engage. Unmarried and first-time mothers may seek extra support during pregnancy. The finding that participants with more baseline social support tended to message more is consistent with established links between psychosocial health and healthcare engagement [35]. Our findings on age and education differ from the WelTel PMTCT study in Kenya, where younger and more educated participants were less responsive to SMS messages [31]. This contrast may be explained by the differing designs of the interventions, as the WelTel tool was not intended for interactive engagement. Higher engagement among more educated participants raises equity concerns, as those with lower education, who are often at higher risk, may have a greater need for accessible information and support.

The associations we found between engagement and maternal-neonatal outcomes are challenging to interpret due to the difficulty in disentangling the direction of causality. For instance, the finding that more messaging is linked to a greater increase in NDS knowledge could be because the messaging directly enhanced knowledge, or it could be that more engaged mothers—who would have increased their knowledge regardless—were simply more active in the intervention. We adjusted for participant demographic characteristics to reduce such confounding, but health-seeking behavior remains an unobserved confounder that is not fully captured by our adjustment variables. The lack of consistent findings around engagement and improved maternal-neonatal outcomes may indicate that the theory of change we proposed: improving knowledge, skills and behaviors was not the primary change needed to improve outcomes.

The association between higher messaging frequency and lower odds of early breastfeeding is an unexpected finding. In exploratory analyses, we found this association only among participants in the Nairobi clinics, suggesting that it may be related to specific early breastfeeding practices in delivery facilities in that region. The vast majority of deliveries (98%) in this study were in facilities, meaning early initiation of breastfeeding took place at the facility and was likely influenced by maternity health care practices such as early separation of mothers and newborns particularly after operative or complicated deliveries. It is possible that mothers with higher risk pregnancies messaged more because of these risks and were also more likely to have complications that led to the newborn being separated from the mother at birth. Another explanation is that mothers who were skeptical about breastfeeding may have messaged the nurses more frequently with follow-up questions about early breastfeeding guidance. However, in qualitative interviews after trial completion, participants were particularly appreciative of the detailed breastfeeding counseling. Similarly, the non-significant trends showing that increased messaging is associated with higher depression and lower social support scores may seem counterintuitive. However, this could indicate that participants facing postpartum psychosocial challenges reached out to study clinicians more frequently for support. It seems unlikely that the intervention itself would increase depressive symptoms or reduce social support.

There are several limitations to our analysis. First, any significant findings in the association analysis should not be interpreted as causal. Indeed, the primary analysis of the trial did not find any causal effects on maternal-neonatal health outcomes [33]. Second, we used a straightforward approach to engagement, focusing on the number of messages sent by participants and the character count of those messages. The messages themselves hold much more information that can be explored. In future analyses, we will delve deeper into the content and topics of the messages. Another potential extension is to use the data to develop a predictive model that could identify if messaging patterns can forecast poor health outcomes, such as hospitalization or death. This approach could help pinpoint participants at the highest risk, which is especially crucial in resource-limited areas.

Our analysis contributes to the field’s understanding of who engages in mHealth interventions, how they engage, and whether engagement is associated with intervention outcomes. This is important for two reasons. First, identifying who engages can help develop targeted strategies to boost participation among groups less likely to engage—such as older or less educated mothers in our study. Tailoring content delivery based on participant characteristics could improve the overall effectiveness of mHealth interventions, particularly if individuals at high risk are less likely to engage. If an intervention were to be adopted in our study population, it would be important to proactively address barriers to engagement among groups less likely to participate—such as older, less educated, or time-constrained mothers. Second, understanding whether increased engagement correlates with better outcomes is key to uncovering the mechanisms of action for the intervention. While the parent trial did not find that Mobile WACh NEO was efficacious, and our analysis did not establish causal mechanisms, our findings offer valuable insights to generate hypotheses for future research. To clarify the directionality of associations, future studies could collect participant proxies for health-seeking behavior and adjust for them. Alternatively, an intervention could be designed to randomize participants to different doses, directly testing whether varying levels of the intervention impact health outcomes. If patterns of engagement are found to be associated with negative outcomes, monitoring engagement metrics in clinical settings could enable earlier identification of at-risk participants and prompt targeted follow-up. Future studies might also explore adaptive mHealth models in which the intensity or delivery mode of support dynamically changes based on participants’ engagement behavior.

mHealth studies should routinely report on engagement metrics and explore their relationship to health outcomes to shed light on possible mechanisms and improve interventions. Despite the growing use of digital health, these aspects are often underexplored. While our study sheds light on both engagement patterns and their links to health outcomes, further research is needed to establish causal pathways and optimize mHealth interventions.

## Supporting information

S1 Table Description of the maternal-neonatal outcomes examined in the association analyses and analytic approach for each outcome, including inclusion criteria, exposure definition, and statistical analysis. (PDF)

S2 Table Table of raw outcomes. For binary outcomes, we display the number and percent with the outcome, and for continuous outcomes, we display the mean and IQR.
(PDF)

S1 Fig Average number of (a) system automated (top panel) and (b) nurse messages (bottom panel) sent by week, relative to the date of delivery. (PDF)

S2 Fig Results of association analyses, unadjusted. The (a) top panel displays the estimated odds ratios for binary outcomes, while (b) the bottom panel presents the estimated differences for continuous outcomes. For early breastfeeding and self-efficacy outcomes, multiple imputation was used to generate the estimates and confidence intervals.
(PDF)

S3 Fig Results of association analyses using normalized participant and nurse message length (per 100 characters) as the exposure. The (a) top panel displays the estimated odds ratios for binary outcomes, while the (b) bottom panel presents the estimated differences for continuous outcomes. For the home provision of KMC outcome, only the unadjusted estimate is shown because only 11 participants had the positive outcome. For early breastfeeding and self-efficacy outcomes, multiple imputation was used to generate the estimates and confidence intervals.
(PDF)
