# Supplementary material for: Associations between text communication engagement and maternal-neonatal outcomes in the Mobile WACh NEO Trial
Source: PLOS Digit Health. 2025 Aug 7;4(8):e0000968. doi: 10.1371/journal.pdig.0000968 (PMC12331090; doi:10.1371/journal.pdig.0000968)
Supplement: S1 Table — (PDF) [file pdig.0000968.s001.pdf]

S1 Table

Description of the maternal-infant outcomes examined in the association analyses and analytic approach for each outcome, including inclusion criteria, exposure definition, and statistical analysis

**Outcome**	**Definition**	**Inclusion criteria**	**Timing**	**Exposure definition**	**Statistical analysis**
Neonatal mortality	Death during 1st 28 days of life	Exclude stillbirths	From delivery until 4 weeks	Messages prior to birth	Logistic regression
Initiation of early breastfeeding	Breastfeeding in 1st hour of life	Exclude stillbirths	First hour of birth	Messages prior to birth	Logistic regression
Exclusive breastfeeding	Cessation of EBF in 1st 6 weeks of life	Exclude stillbirths and infant deaths	At 6 weeks	Messages across entire study period	Logistic regression with multiple imputation
Thermal care	Bath in first 24 hours of life	Exclude stillbirths and infant deaths on day of delivery	First 24 hours after birth	Messages prior to birth	Logistic regression
Home provision of KMC	Any duration of skin-to-skin care on ≥10 of the first 14 days at home, among low birthweight or preterm infants	Low birthweight and preterm infants only; exclude stillbirths, infants who passed away in the facility having never left the facility, and infant deaths at 14 days or younger	At 2 weeks	Messages up to 2 weeks	Logistic regression
Cord care	No application of substances to cord	Exclude stillbirths	At 2 weeks	Messages up to 2 weeks	Logistic regression

Maternal knowledge of neonatal danger signs (NDS)	Number of the 8 danger signs or symptoms successfully named (0-8)	No exclusions	At 6 weeks	Messages across entire study period	Linear regression on change in NDS identified
Hospitalization	Hospitalization of infant over study period	Exclude stillbirths	From delivery until 6 weeks	Messages prior to hospitalization (for those whose infant was hospitalized); otherwise messages across the entire period	Logistic regression
Depression	Edinburgh Postnatal Depression Scale (0-30)	No exclusions	At 6 weeks	Messages across entire study period	Linear regression on change in score
Social support	Medical Outcomes Study (MOS) Social Support Survey, standardized to the 0-1 scale	No exclusions	At 6 weeks	Messages across entire study period	Linear regression on change in score
Self-efficacy	Score using Karitane Parenting Confidence Scale (0-45)	No exclusions	At 6 weeks	Messages across entire study period	Linear regression on change in score with multiple imputation
