# Supplementary material for: Associations between text communication engagement and maternal-neonatal outcomes in the Mobile WACh NEO Trial
Source: PLOS Digit Health. 2025 Aug 7;4(8):e0000968. doi: 10.1371/journal.pdig.0000968 (PMC12331090; doi:10.1371/journal.pdig.0000968)
Supplement: S2 Table — For binary outcomes, we display the number and percent with the outcome, and for continuous outcomes, we display the mean and IQR. (PDF) [file pdig.0000968.s002.pdf]

S2 Table

Table of raw outcomes. For binary outcomes, we display the number and percent with the outcome, and for continuous outcomes, we display the mean and IQR.

Outcome	Met inclusion criteria (%)[1]	Ascertained outcome (%)	Number with the event (%) OR mean score change (IQR)
Infant deaths	2437 (98.7%)	2436 (99.9%)	45 (1.8%)
Initiated early breastfeeding	2437 (98.7%)	2082 (85.4%)	1507 (72.4%)
Has been fed food other than breastmilk	2384 (96.5%)	2384 (100%)	195 (8.2%)
Performed thermal care	2422 (98.1%)	2327 (96.1%)	2240 (96.3%)
Home provision of KMC	173 (7.0%)	173 (100%)	11 (6.4%)
Performed correct cord care	2437 (98.7%)	2369 (97.2%)	1119 (47.2%)
Hospitalization	2437 (98.7%)	2437 (100%)	97 (4%)
Change in maternal knowledge of neonatal signs from baseline to 6 weeks postpartum	2470 (100%)	2388 (96.7%)	0.852 (0, 2)
Change in depression score from baseline to 6 weeks postpartum	2470 (100%)	2460 (99.6%)	-0.391 (-2, 1)
Change in social support score from baseline to 6 weeks postpartum	2470 (100%)	2466 (99.8%)	0.177 (-0.316, 0.842)
Change in self-efficacy score from baseline to 6 weeks postpartum	2470 (100%)	1925 (77.9%)	0.705 (-1, 2)

[1] Denominator is the number of intervention participants included in the analysis (2,470).
