# Supplementary material for: Associations between text communication engagement and maternal-neonatal outcomes in the Mobile WACh NEO Trial
Source: PLOS Digit Health. 2025 Aug 7;4(8):e0000968. doi: 10.1371/journal.pdig.0000968 (PMC12331090; doi:10.1371/journal.pdig.0000968)

S1 Fig

Average number of (a) system automated (top panel) and (b) nurse messages (bottom panel) sent by week, relative to the date of delivery.

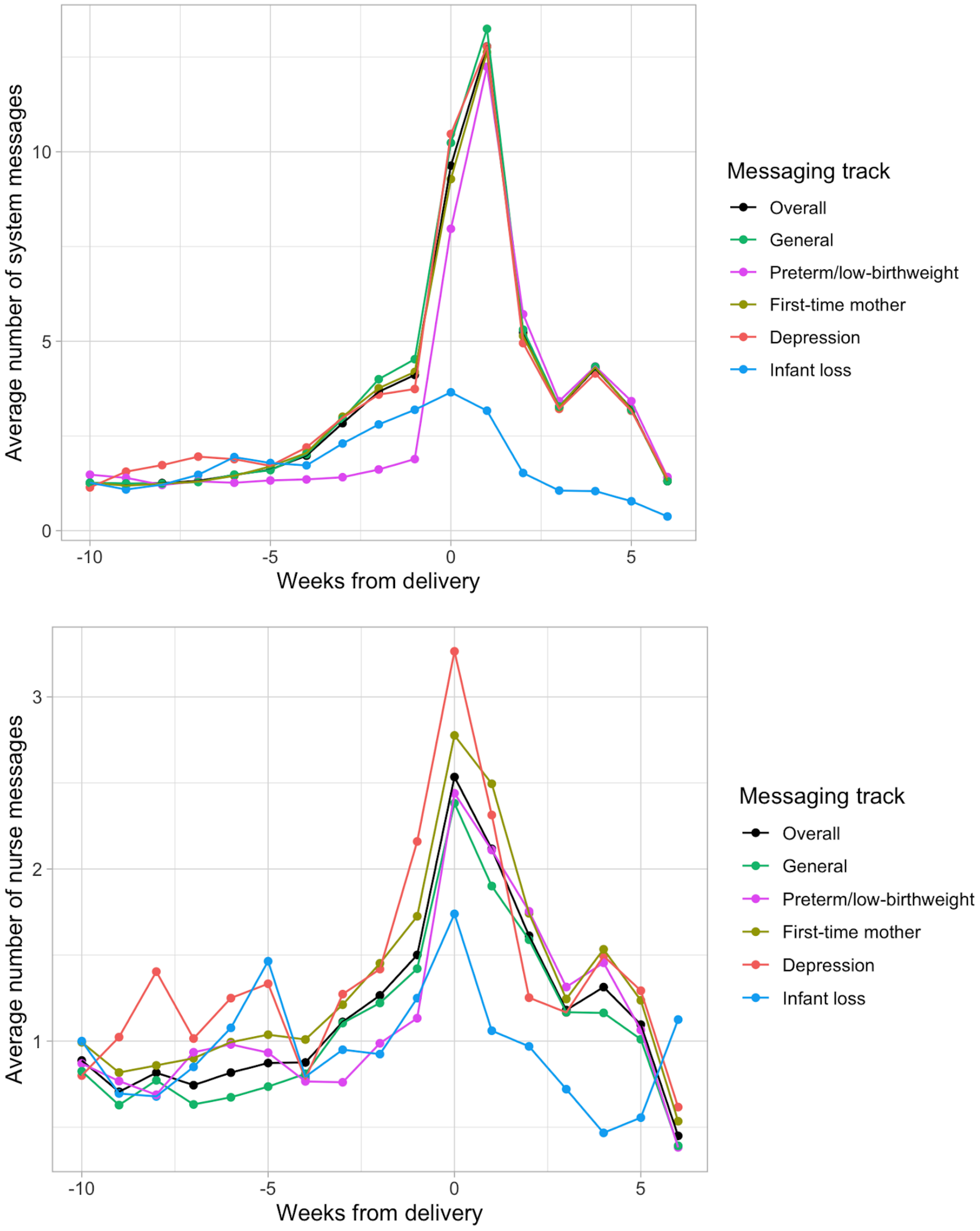

Supplement: S1 Fig — (PDF) [file pdig.0000968.s003.pdf]
