# Supplementary material for: Associations between text communication engagement and maternal-neonatal outcomes in the Mobile WACh NEO Trial
Source: PLOS Digit Health. 2025 Aug 7;4(8):e0000968. doi: 10.1371/journal.pdig.0000968 (PMC12331090; doi:10.1371/journal.pdig.0000968)

S2 Fig

Results of association analyses, unadjusted. The (a) top panel displays the estimated odds ratios for binary outcomes, while (b) the bottom panel presents the estimated differences for continuous outcomes. For early breastfeeding and self-efficacy outcomes, multiple imputation was used to generate the estimates and confidence intervals.

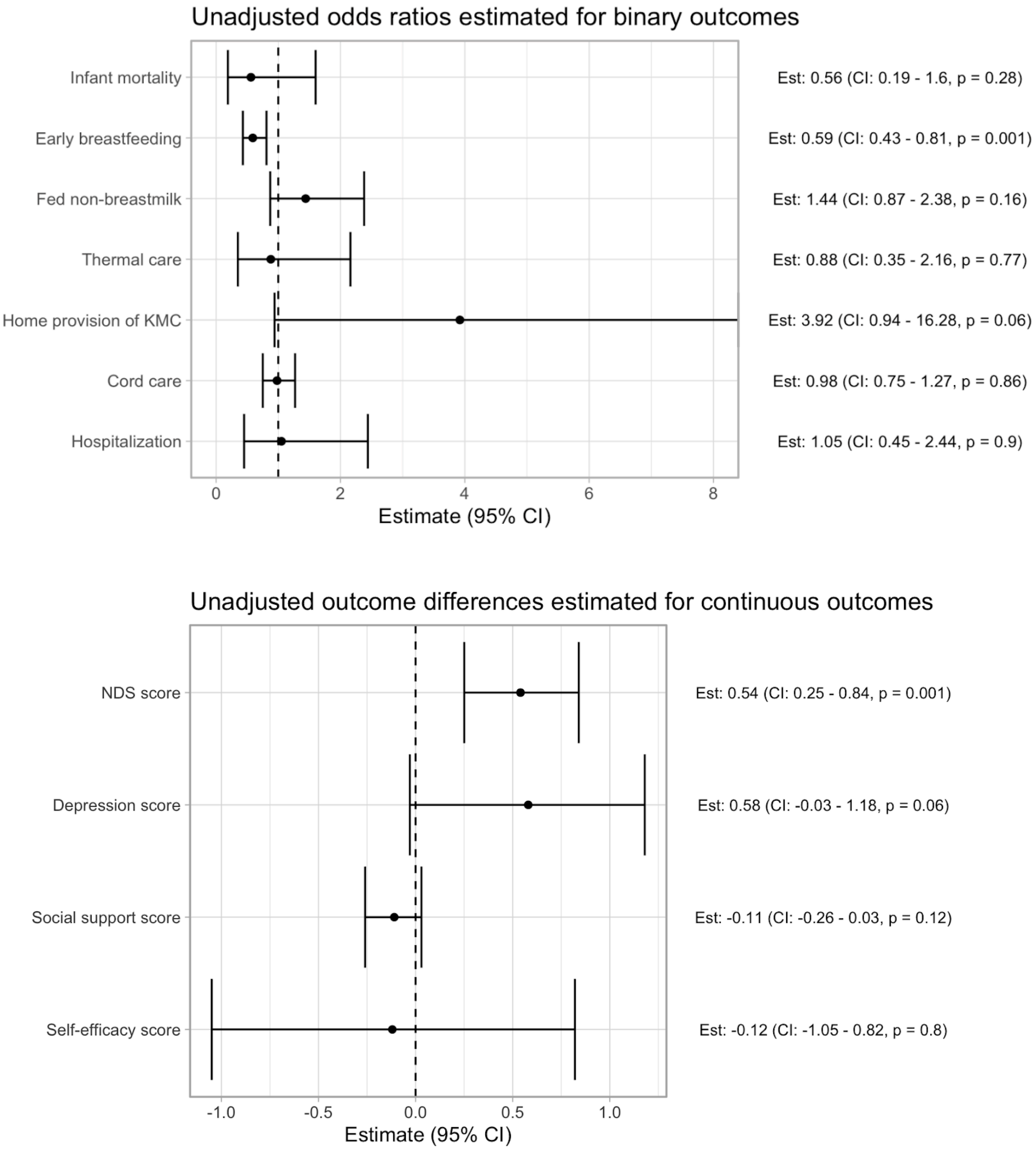

Supplement: S2 Fig — The (a) top panel displays the estimated odds ratios for binary outcomes, while (b) the bottom panel presents the estimated differences for continuous outcomes. For early breastfeeding and self-efficacy outcomes, multiple imputation was used to generate the estimates and confidence intervals. (PDF) [file pdig.0000968.s004.pdf]
