# Supplementary material for: Associations between text communication engagement and maternal-neonatal outcomes in the Mobile WACh NEO Trial
Source: PLOS Digit Health. 2025 Aug 7;4(8):e0000968. doi: 10.1371/journal.pdig.0000968 (PMC12331090; doi:10.1371/journal.pdig.0000968)

S3 Fig

Results of association analyses using normalized participant and nurse message length (per 100 characters) as the exposure. The (a) top panel displays the estimated odds ratios for binary outcomes, while the (b) bottom panel presents the estimated differences for continuous outcomes. For the home provision of KMC outcome, only the unadjusted estimate is shown because only 11 participants had the positive outcome. For early breastfeeding and self-efficacy outcomes, multiple imputation was used to generate the estimates and confidence intervals.

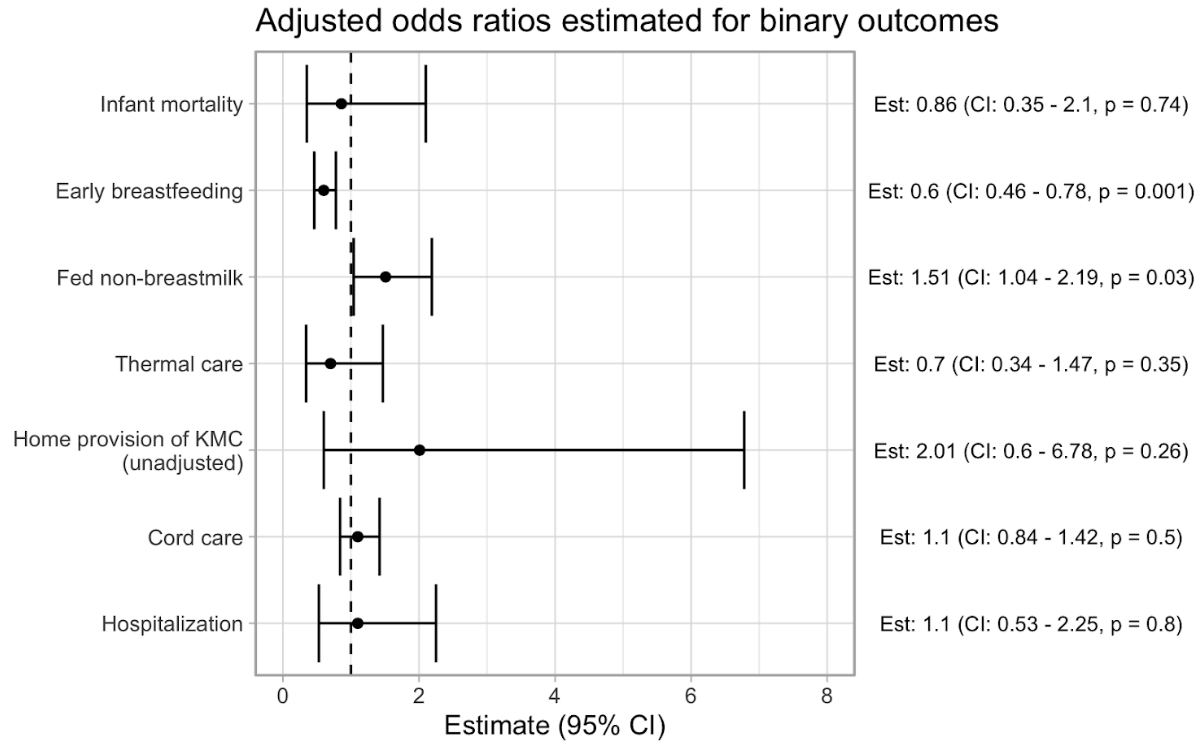

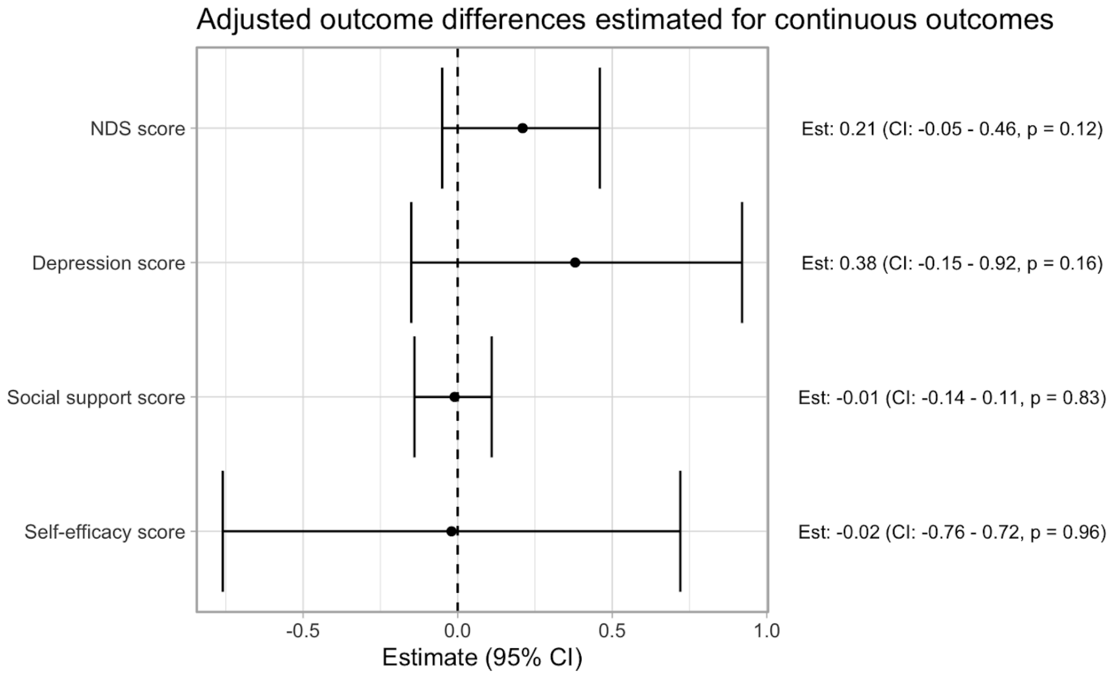

Supplement: S3 Fig — The (a) top panel displays the estimated odds ratios for binary outcomes, while the (b) bottom panel presents the estimated differences for continuous outcomes. For the home provision of KMC outcome, only the unadjusted estimate is shown because only 11 participants had the positive outcome. For early breastfeeding and self-efficacy outcomes, multiple imputation was used to generate the estimates and confidence intervals. (PDF) [file pdig.0000968.s005.pdf]
